# Response of Human Mesenchymal Stromal Cells from Periodontal Tissue to LPS Depends on the Purity but Not on the LPS Source

**DOI:** 10.1155/2020/8704896

**Published:** 2020-07-02

**Authors:** Christian Behm, Alice Blufstein, Setareh Younes Abhari, Christoph Koch, Johannes Gahn, Christina Schäffer, Andreas Moritz, Xiaohui Rausch-Fan, Oleh Andrukhov

**Affiliations:** ^1^Division of Conservative Dentistry and Periodontology, University Clinic of Dentistry, Medical University of Vienna, Vienna, Austria; ^2^Department of NanoBiotechnology/NanoGlycobiology Unit, University of Natural Resources and Life Sciences, Vienna, Austria

## Abstract

Human periodontal ligament stromal cells (hPDLSCs) and gingival mesenchymal stromal cells (hGMSCs) are resident mesenchymal stromal cells (MSCs) of the periodontal tissue. The lipopolysaccharide (LPS) from *Porphyromonas gingivalis* is structurally distinct from that of other Gram-negative bacteria, and earlier studies linked this structural difference to a distinct virulence activity and the ability to activate toll-like receptor 2 (TLR-2), besides TLR-4 as commonly occurring upon LPS challenge. Later studies, in contrast, argue that TLR-2 activation by *P. gingivalis* LPS is due to lipoprotein contamination. In the present study, we aimed to define the influence of structure versus purity of *P. gingivalis* LPS on the immune response of hPDLSCs and hGMSCs. Cells were stimulated with commercially available “standard” *P. gingivalis* LPS, “ultrapure” *P. gingivalis* LPS, or “ultrapure” *Escherichia coli* LPS, and the expression of interleukin- (IL-) 8, IL-6, monocyte chemoattractant protein- (MCP-) 1, TLR-2, and TLR-4 was evaluated. The contribution of TLR-4 to the LPS-induced response was assessed using the specific TLR-4 inhibitor TAK-242. “Standard” *P. gingivalis* LPS induced significantly higher IL-8, IL-6, and MCP-1 production compared to the “ultrapure” LPS preparations, with no significant difference detectable for “ultrapure” LPS from *P. gingivalis* and *E. coli*. By using TAK-242, the response of hPDLSCs and hGMSCs to “ultrapure” LPS preparations was effectively inhibited to the levels comparable to those of nonstimulated controls. In contrast, high levels of response to “standard” LPS were observed, even in the presence of TAK-242. Our data show that the response of MSCs from periodontal tissue to LPS depends more on the purity of the LPS preparation than on the LPS source. Even a small amount of contaminating lipoproteins can drastically enhance the hPDLSCs' and hGMSCs; responsiveness to *P. gingivalis* LPS, which might also contribute to the progression of periodontal disease.

## 1. Introduction

Human periodontal ligament stromal cells (hPDLSCs) and human gingiva-derived mesenchymal stromal cells (hGMSCs) isolated from periodontal ligament [1] and the gingiva [2], respectively, fulfil the minimal criteria of mesenchymal stromal cells (MSCs) [3] and have characteristics comparable to bone marrow-derived MSCs [4]. Both cell types influence immune and inflammatory responses, by acting either as immunosuppressors, mainly by producing immunomediators, or as immunostimulators, by secreting various proinflammatory mediators [5, 6]. MSCs from periodontal tissue reside in the perivascular area and, therefore, might directly interact with immune cells during their transendothelial migration. Further, they can migrate into inflamed or regenerating tissue upon sensing different chemoattractant stimuli [5, 7, 8]. hGMSCs and hPDLSCs comprise diverse functions such as regulating periodontal tissue homeostasis and regeneration and inflammatory responses in periodontal disease progression, which pinpoints a potential use of these cells as therapeutic tool for oral and extraoral tissue regeneration [7–12].

Periodontitis is an inflammatory, multifactorial, chronic disease of polymicrobial etiology, causing the destruction of the periodontium which, in worst cases, leads to tooth loss [13, 14]. The main reason for periodontitis is the overgrowth of distinct Gram-negative bacteria, leading to the disruption of the bacteria-host homeostasis and resulting in an inappropriate, overwhelming immune response. The Gram-negative bacterium *Porphyromonas gingivalis* is a keystone pathogen that is strongly associated with periodontitis [15]. Lipopolysaccharide (LPS), a crucial virulence factor of *P. gingivalis* [16], induces the production of several proinflammatory mediators like interleukin- (IL-) 1*β*, IL-6, IL-8, tumor necrosis factor alpha (TNF-*α*), and monocyte chemoattractant protein 1 (MCP-1) by hPDLSCs [17–19] and hGMSCs [20–22]. Production of these mediators contributes to an excessive inflammatory response, leading to the destruction of the periodontal tissue and to alveolar bone resorption [23].

LPS is a characteristic component of the outer membrane of Gram-negative bacteria [24] and leads to a potent proinflammatory immune response in various cell types [25]. It is recognized by toll-like receptor- (TLR-) 4 [26–28], which is known to be expressed in hPDLSCs and hGMSCs, together with TLR-2 and other TLR family members [29, 30]. The structure of lipid A from *P. gingivalis* LPS differs from that of most Gram-negative bacteria. This is thought to result in distinct virulence activities and even the ability to activate TLR-2, [26, 31], a known cell receptor of bacterial lipoproteins and peptidoglycan [32]. Furthermore, lipid A of *P. gingivalis* LPS exhibits a certain degree of heterogeneity in fatty acids, which can influence the inflammatory response [33] and exhibit certain heterogeneity, which might result in differences in the inflammatory response. There are indications that components that have been coisolated during LPS preparation could be the reason for this ambiguity. An early study of Hirschfeld et al. showed that removal of lipoproteins from “standard” LPS results in abolishment of the TLR-2 response [34]. Another study demonstrated that lipoproteins in “standard” *P. gingivalis* LPS preparations could be potent TLR-2 activators [35]. Above that, Ogawa et al. showed that synthetic *P. gingivalis* lipid A activates TLR-4 only [36].

Currently, there is no study available that compares the inflammatory response of hPDLSCs and hGMSCs to LPS preparations of different sources and purity. *P. gingivalis* LPS preparations are commercially available in two grades—“standard” *P. gingivalis* LPS, isolated from bacteria by phenol-water extraction [37], and “ultrapure” *P. gingivalis* LPS. “Standard” LPS preparations are known to contain traces of lipoproteins [38] which influence the host response [34, 39], while “ultrapure” LPS that is additionally treated with enzymes to degrade lipoproteins was shown to no longer activate TLR-2 reporter HEK-Blue hTLR2-hCD14 cells [38]. The same study showed that “standard” *P. gingivalis* LPS activates cytokine production in macrophages through both TLR-2 and TLR-4, whereas “ultrapure” LPS acts exclusively through TLR-4 activation [39]. Since *P. gingivalis* LPS is considered an important factor in periodontal disease pathogenesis, it is important to understand its effect on different cells from the periodontal tissue and discriminate the contribution of its purity versus structural features to the host response in MSCs of periodontal tissue.

In the present study, we compared *in vitro* the response of MSCs from periodontal tissues to commercially available “standard” *P. gingivalis* LPS, “ultrapure” *P. gingivalis* LPS, and “ultrapure” *E. coli* LPS. The involvement of TLR-4 in the response to the different LPS preparations was assessed by blocking TLR-4 with the TLR-4 specific inhibitor TAK-242.

## 2. Material and Methods

### 2.1. Compositional Analysis of *P. gingivalis* LPS Preparations

*P. gingivalis* LPS preparations of different purity were purchased from Invivogen (San Diego, USA); these are “standard” *P. gingivalis* LPS, extracted from bacteria with phenol-water, and “ultrapure” *P. gingivalis* LPS. Both *P. gingivalis* LPS preparations were obtained by a similar proprietary procedure, except for the additional treatment of “ultrapure” LPS with proteolytic enzymes for lipoprotein degradation, according to the manufacturer's specifications. The following commercially available LPS (Invivogen, San Diego, USA) were used within the frame of this study: “standard” *P. gingivalis* LPS (Cat. No. tlrl-pglps), “ultrapure” *P. gingivalis* LPS (Cat. No. tlrl-ppglps), and “ultrapure” *E. coli* LPS (Cat. No. tlrl-pb5lps).

To provide a measure of lipoprotein contamination versus LPS content, the LPS preparations were analyzed for the monosaccharides constituting *P. gingivalis* A-LPS [40, 41] and O-LPS [42], including 3-deoxy-*D-*manno-oct-2-ulosonic acid (KDO), which is a common constituent of the LPS core. Monosaccharides were quantified after hydrolysis of 250 *μ*g of LPS with 200 *μ*l of trifluoroacetic acid for 4 h at 110 C by high-performance anion-exchange chromatography with pulsed amperometric detection (HPAEC-PAD) on a PA-1 column (ICS3000 chromatographic system equipped with an Electrochemical Detector ED3000; Dionex Austria GmbH, Thermo Fisher Scientific), using authentic standards. The following eluents were used at a flow rate of 1 ml/min: (A), Aqua dest., (B) 100 mM NaOH, and (C) 1 M sodium acetate in 100 mM NaOH. The gradient was 0-21 min: 84.8% A/15% B/0.2% C; 21-27 min: 0% A/98.8% B/0.2% C; 27-59 min: 0% A/68.0% B/32.0% C; 59-65 min: 0% A/98.8% B/0.2% C; 55-68 min: 84.8% A/15.0% B/0.2% C; and 68-80 min: 84.8% A/1.0% B/0.2% C.

The protein concentration was determined spectrophotometrically at 280 nm using a NanoDrop device. To visualize lipoproteins, SDS gel electrophoresis of 1, 2, 5, and 10 *μ*g of “standard” and “ultrapure” LPS was carried out on 8-16% Tris/Tricine TGX precast gels (Bio-Rad, Vienna, Austria) in a Mini-Protean electrophoresis apparatus (Bio-Rad), followed by silver staining [43].

### 2.2. Cell Culture

Primary hPDLSCs and hGMSCs were isolated from third molar teeth from five different periodontally healthy patients as described in our previous study [18]. The third molar teeth were extracted due to orthodontic reasons. The study protocol was approved by the Ethics Committee of the Medical University of Vienna (EK-Nr. 1694/2015). The methods were carried out in accordance with the relevant guidelines and regulations; all patients got informed before the surgical procedure and gave their written consent. Cells were cultured in Dulbecco's modified Eagle's medium (DMEM, Sigma-Aldrich, St. Louis, USA), supplemented with 10% fetal bovine serum (FBS, Gibco, Carlsbad, USA), 50 *μ*g/ml streptomycin (S), and 100 U/ml penicillin (P) (Gibco, Carlsbad, USA). Cells from passages 3 to 7 were used for all experiments.

### 2.3. Verification of MSC Surface Marker Expression on hPDLSCs and hGMSCs

hPDLSCs and hGMSCs were characterized by analyzing the expression of characteristic cell surface markers. Single cell suspensions were stained 1 : 10 with one of the following antibodies (all from eBioscience, San Diego, USA) for 20 min: phycoerythrin- (PE-) conjugated mouse anti-human CD90, PE-conjugated mouse anti-human CD105, PE-conjugated mouse anti-human CD146, PE-conjugated mouse anti-human CD29, PE-conjugated mouse anti-human CD73, fluorescein isothiocyanate- (FITC-) conjugated mouse anti-human CD34, FITC-conjugated mouse anti-human CD45, and FITC-conjugated mouse anti-human CD31. After resuspending cells in 200 *μ*l of FACS buffer (3% BSA, 0.09% sodium azide, in 1x PBS), an argon laser was used, exciting the fluorescence at 488 nm. The percentage of positive cells for each investigated surface marker was determined using the FACScan Flow Cytometer (Becton Dickinson, Franklin Lakes, USA).

### 2.4. Stimulation Protocol

5 × 10^4^ cells were seeded per well in 24-well plates, in 0.5 ml DMEM, supplemented with 10% FBS and 1% P/S. After 24 h of incubation, the medium was changed to FBS-free DMEM, supplemented with 1% P/S, and cells were stimulated with the different LPS preparations for 4 or 24 h. All three preparations—“standard” *P. gingivalis* LPS, “ultrapure” *P. gingivalis* LPS, and “ultrapure” *E. coli* LPS—were dissolved at a concentration of 1 mg/ml in endotoxin-free water. Stimulation was performed in the presence of 1 *μ*g/ml LPS and in the presence of exogenous soluble (s)CD14 (250 ng/ml, Sigma-Aldrich, St. Louis, USA). In one series of experiments, TLR-4 was inhibited by TAK-242 (Cayman Chemical, Ann Arbor, USA). In these experiments, cells were pretreated with 5 *μ*M of TAK-242 in FBS-free medium for 1 h prior to stimulation and 5 *μ*M TAK-242 was supplemented during the whole stimulation time. All stimulations were performed in duplicate. Cell viability was measured by the MTT method. IL-8, IL-6, MCP-1, TLR-2, and TLR-4 gene expression levels were determined using quantitative polymerase chain reaction (qPCR). The protein levels of IL-6, IL-8, and MCP-1 were measured in conditioned media, using enzyme-linked immunosorbent assay (ELISA).

### 2.5. Cell Viability

Cell viability was measured similarly to a previously described method [44]. Briefly, 100 *μ*l of 3,4,5-dimethylthiazol-2-yl-2,5-diphenyl tetrazolium bromide (MTT) reagent (5 mg/ml) (Sigma-Aldrich, St. Louis, USA) was added to each well at the end of stimulation followed by an incubation at 37° C for two hours. Subsequently, the medium was discarded and 500 *μ*l dimethylsulfoxide was added to each well to dissolve formed formazan crystals. 100 *μ*l of the dissolved crystals were transferred into 96-well plates in quadruplets, and the absorbance was measured at 570 nm using a microplate reader (Synergy HTX multiplate reader, BioTek, USA).

### 2.6. Quantitative PCR

Cell lysis, total cellular mRNA extraction, cDNA synthesis, and qPCR were performed using TaqMan Gene expression Cells-to-CT kit (Applied Biosystems, Foster City, USA), according to the manufacturer's protocol. qPCR was conducted in paired reactions using an ABI StepOnePlus device (Applied Biosystems, Foster City, USA), and the following TaqMan Gene Expression Assays (Applied Biosystems, Foster City, USA) were used: IL-6, Hs00985639_m1; IL-8, Hs00174103; MCP-1, Hs00234140_m1; TLR-2, Hs00610101_m1; TLR-4, Hs00152939_m1; and GAPDH, Hs99999905. The following thermocycler settings were used: once 95°C for 10 min followed by 50 cycles, each consisting of 15 s at 95°C and 1 minute at 60°C. For each sample, the *C*_t_ value was determined. The relative expression of the target genes compared to untreated control was calculated, using the 2^-∆∆Ct^ method, using GAPDH as internal reference.

### 2.7. Enzyme-Linked Immunosorbent Assay

Conditioned media were harvested, followed by determining IL-8, IL-6, and MCP-1 protein levels. ELISA Ready-Set-Go! Kits (eBioscience, Waltham, USA) were used, according to the manufacturers' protocols. The concentrations of the standards provided in the kits ranged between 2 and 200 pg/ml for IL-6, 2 and 250 pg/ml for IL-8, and 7 and 1000 pg/ml for MCP-1. ELISAs were performed in duplicate per group, followed by measuring the optical density at 450 nm (OD_450_). Concentrations were calculated by plotting the measured OD_450_ values against the appropriate standard curves.

### 2.8. Statistical Analysis

All statistical analysis was performed using the statistical program SPSS 24.0 (IBM, Armonk, USA). The difference between different groups was tested by the Friedman test, followed by the Wilcoxon test for pairwise comparison. Statistical differences showing *P* values < 0.05 were considered statistically significant. All data are expressed as mean values ± standard error of mean (s.e.m.), from five independent experiments from five different donors.

## 3. Results

### 3.1. Composition of Commercial *P. gingivalis* LPS Preparations

According to monosaccharide analysis by HPAEC-PED, both “standard” and “ultrapure” LPS contain the known monosaccharide constituents of *P. gingivalis* A-LPS and O-LPS [40, 42]. These are rhamnose, *N*-acetylgalactosamine, *N*-acetylglucosamine, galactose, glucose, mannose, and KDO in a molar ratio of 0.6/0.6/0.6/1.0/1.3/0.2/<0.1, with galactose arbitrarily set to 1.0. The overall sugar content of the purchased “1 mg/ml” LPS preparations was increased (by 38.0%) in the “ultrapure” LPS preparation in comparison to “standard” LPS, with 24.8% (*w*/*w*) versus 17.8% (*w*/*w*).

According to protein measurement with the NanoDrop device, the protein concentration of the “ultrapure” LPS preparation in comparison to “standard” LPS was increased twofold. This approximates also the enrichment of the LPS in the “ultrapure” preparation. To learn more about the composition of the two LPS samples, we next performed an SDS gel electrophoresis. On a silver-stained Tris/Tricine gradient gel, either preparation revealed a ladder-like banding pattern typical of LPS, with an evident enrichment of LPS of longer O-polysaccharide chains in the “ultrapure” LPS preparation. In the “standard” LPS preparation, five distinct bands migrating within a molecular weight range between below 10 kDa and 15 kDa could be visualized in a concentration-dependent manner. These bands were clearly missing in the “ultrapure” LPS sample (Supplementary Figure 1). Based on a previous study by others, these latter bands are likely representing *P. gingivalis* lipoproteins [35].

### 3.2. Mesenchymal and Hematopoietic Surface Marker Expression in hPDLSCs and hGMSCs

hPDLSCs as well as hGMSCs were stained positively (<95%, except CD146) for all investigated MSC surface markers (CD90, CD29, CD105, CD146, and CD73, Supplementary Table 1). Additionally, both cell types were stained negatively (<3%) for the investigated hematopoietic surface markers (CD31, CD34, and CD45, Supplementary Table 1).

### 3.3. Viability of hPDLSCs and hGMSCs in Response to Stimulation with Different LPS Preparations

**Figure 1:**
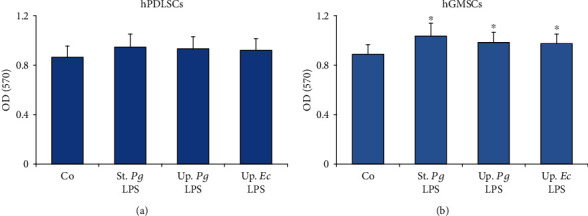
Viability of hPDLSCs and hGMSCs in response to stimulation with different LPS preparations. Primary hPDLSCs and hGMSCs were stimulated with commercially available “standard” *P. gingivalis* LPS (1 *μ*g/ml), “ultrapure” *P. gingivalis* LPS (1 *μ*g/ml), and “ultrapure” *E. coli* LPS (1 *μ*g/ml) in the presence of 250 ng/ml sCD14 for 24 hours. Cell viability was assessed by the MTT method. The *y*-axis shows OD values (570 nm) presented as mean ± s.e.m. of five different donors. ^∗^Significantly different *vs.* control.

The effect of different LPS preparations on the viability of hPDLSCs and hGMSCs is shown in Figure 1. In hPDLSCs, none of the LPS preparations had an effect on cell viability. In hGMSCs, cell viability was slightly increased by all LPS preparations. In both cell types, no differences in cell viability after stimulation with different LPS preparations were observed.

### 3.4. Response of Primary hPDLSCs and hGMSCs to Different LPS Preparations

**Figure 2:**
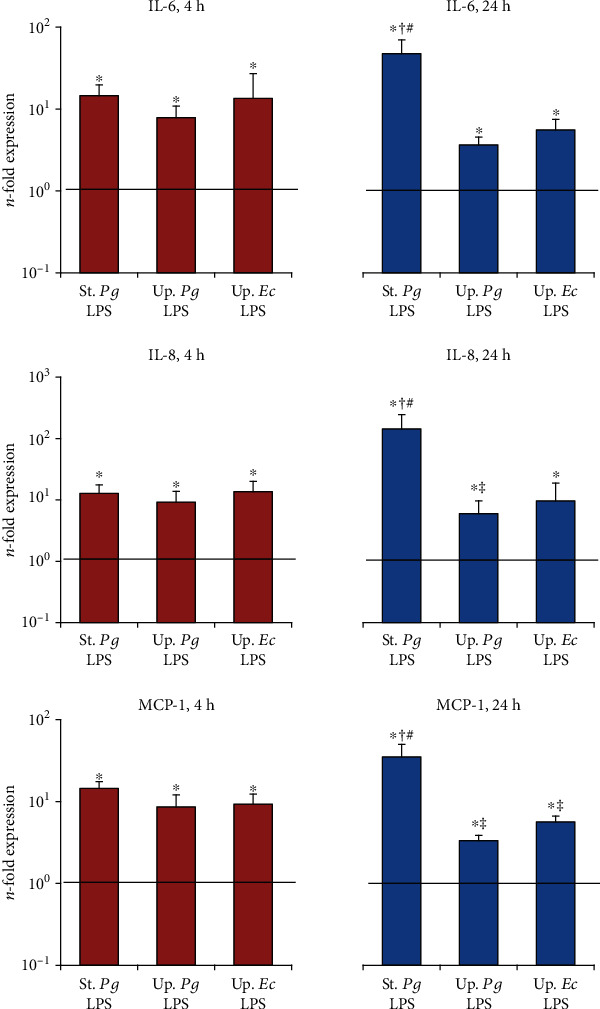
Effect of different *P. gingivalis* LPS preparations on gene expression of IL-6, IL-8, and MCP-1 in hPDLSCs. Primary hPDLSCs were stimulated with commercially available “standard” *P. gingivalis* LPS (1 *μ*g/ml), “ultrapure” *P. gingivalis* LPS (1 *μ*g/ml), and “ultrapure” *E. coli* LPS (1 *μ*g/ml) in the presence (250 ng/ml) of sCD14 for 4 or 24 h. Gene expression levels of IL-6, IL-8, and MCP-1 were measured by qPCR. The *y*-axis represents the *n*-fold expression levels of the target gene in relation to unstimulated cells (*n* = 1). Data are presented as mean ± s.e.m. of five different donors. ^∗^Significantly different *vs.* control; ^#^significantly higher *vs.* “ultrapure” LPS preparations; ^†^significantly higher compared to 4 h of stimulation; ^‡^significantly lower compared to 4 h of stimulation.

**Figure 3:**
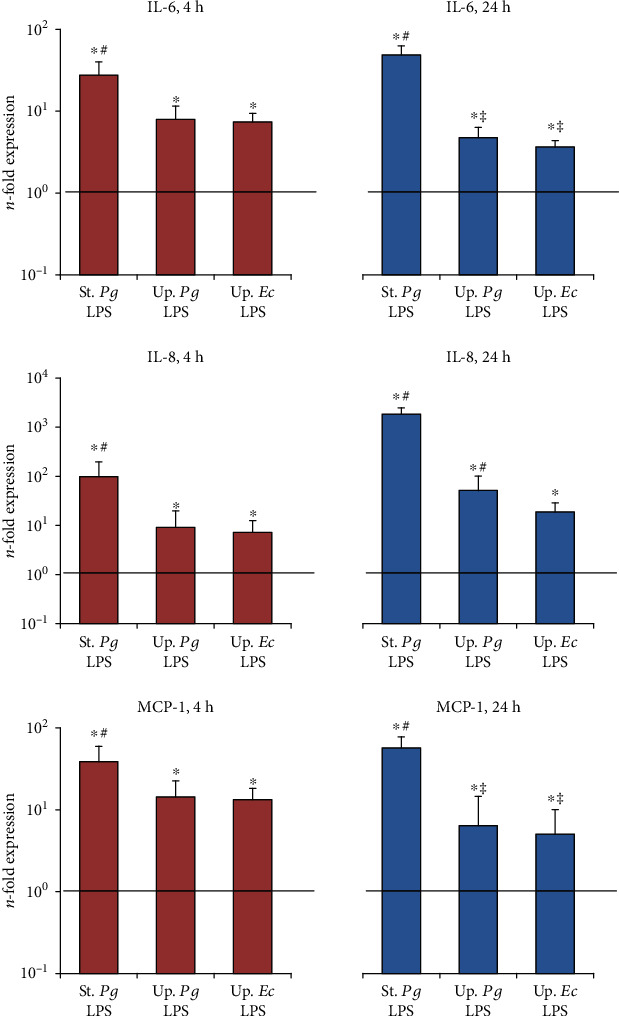
Effect of different *P. gingivalis* LPS preparations on the gene expression of IL-6, IL-8, and MCP-1 in hGMSCs. Primary hGMSCs were stimulated with commercially available “standard” *P. gingivalis* LPS (1 *μ*g/ml), “ultrapure” *P. gingivalis* LPS (1 *μ*g/ml), and “ultrapure” *E. coli* LPS (1 *μ*g/ml) in the presence (250 ng/ml) of sCD14 for 4 or 24 h. Gene expression levels of IL-6, IL-8, and MCP-1 were measured by qPCR. The *y*-axis represents the *n*-fold expression levels of target gene in relation to unstimulated cells (*n* = 1). Data are presented as mean ± s.e.m. of five different donors. ^∗^Significantly different *vs.* control; ^#^significantly higher *vs.* “ultrapure” LPS preparations; ^†^significantly higher compared to 4 h of stimulation; ^‡^significantly lower compared to 4 h of stimulation.

The effect of the different LPS stimuli on the gene expression of IL-6, IL-8, and MCP-1 in primary hPDLSC and hGMSCs after different stimulation times is shown in Figures 2 and 3, respectively. In hPDLSCs, no significant difference was observed between the responses to the different LPS preparations after 4 h of stimulation, while after 24 h of stimulation, the gene expression levels of all tested proteins were significantly higher for “standard” *P. gingivalis* LPS compared to both “ultrapure” LPS. Further, the gene expression levels of IL-8 upon stimulation with “ultrapure” *P. gingivalis* LPS and of MCP-1 upon stimulation with “ultrapure” LPS from both *E. coli* and *P. gingivalis* after 24 h stimulation were significantly lower compared to those after 4 h of stimulation. In hGMSCs, a significantly higher response to “standard” *P. gingivalis* LPS than to “ultrapure” LPS was observed after both 4 h and 24 h. Stimulation of hGMSCs with both “ultrapure” LPS preparations for 24 h caused significantly lower IL-6 and MCP-1 expression levels compared to that for 4 h. In both cell types, no difference between the response to “ultrapure” LPS from *P. gingivalis* and *E. coli* was detected.

**Figure 4:**
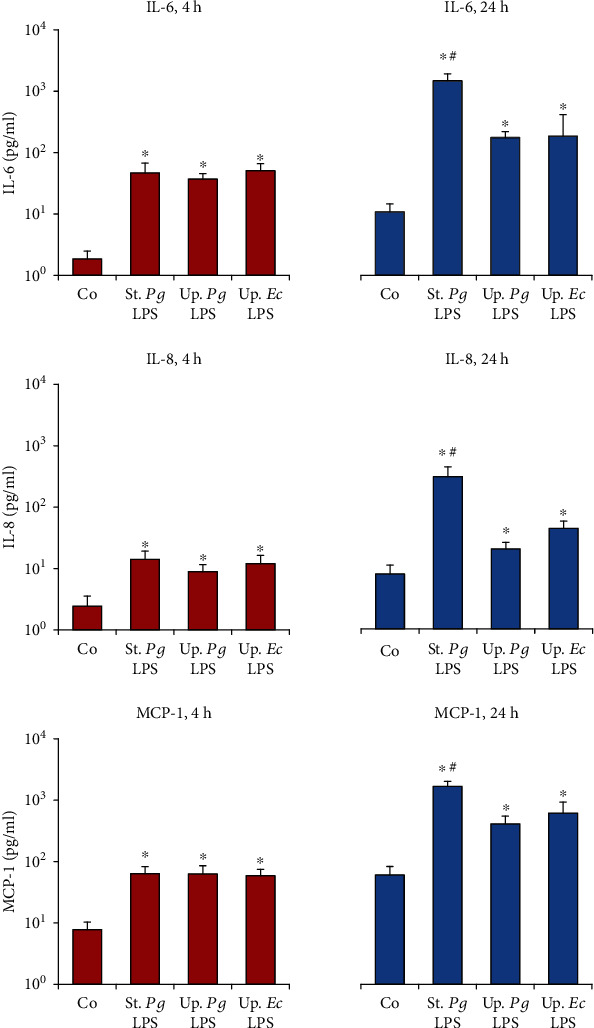
Effect of different *P. gingivalis* LPS preparations on the concentrations of IL-6, IL-8, and MCP-1 in hPDLSC-conditioned media. Primary hPDLSCs were stimulated with commercially available “standard” *P. gingivalis* LPS (1 *μ*g/ml), “ultrapure” *P. gingivalis* LPS (1 *μ*g/ml), and “ultrapure” *E. coli* LPS (1 *μ*g/ml) in the presence (250 ng/ml) of sCD14 for 4 or 24 h. The concentrations of IL-6, IL-8, and MCP-1 in conditioned media were measured by ELISA. Data are presented as mean ± s.e.m. of five different donors. ^∗^Significantly different *vs.* control; ^#^significantly higher *vs.* “ultrapure” LPS preparations; ^†^significantly higher compared to 4 h of stimulation; ^‡^significantly lower compared to 4 h of stimulation.

**Figure 5:**
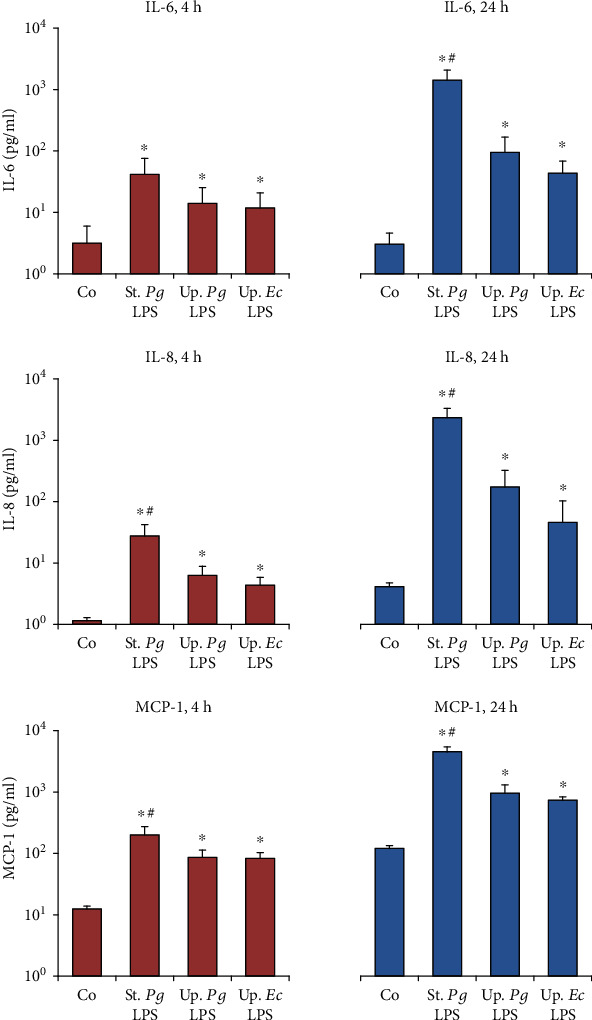
Effect of different *P. gingivalis* LPS preparations on concentrations of IL-6, IL-8, and MCP-1 in hGMSC-conditioned media. Primary hGMSCs were stimulated with commercially available “standard” *P. gingivalis* LPS (1 *μ*g/ml), “ultrapure” *P. gingivalis* LPS (1 *μ*g/ml), and “ultrapure” *E. coli* LPS (1 *μ*g/ml) in the presence (250 ng/ml) of sCD14 for 4 or 24 h. The concentrations of IL-6, IL-8, and MCP-1 in conditioned media were measured by ELISA. Data are presented as mean ± s.e.m. of five different donors. ^∗^Significantly different *vs.* control; ^#^significantly higher *vs.* “ultrapure” LPS preparations; ^†^significantly higher compared to 4 h of stimulation; ^‡^significantly lower compared to 4 h of stimulation.

Next, we determined the concentrations of IL-6, IL-8, and MCP-1 in conditioned media of hPDLSCs and hGMSCs upon stimulation with the different LPS preparations (Figures 4 and 5). In hPDLSCs, the concentration of all proinflammatory mediators in response to “standard” *P. gingivalis* LPS was significantly higher than that to both “ultrapure” LPS preparations after 24 h, but not after 4 h. In hGMSCs “standard” *P. gingivalis* LPS induced a significantly higher IL-8 and MCP-1 concentration compared to “ultrapure” LPS after 4 and 24 h of stimulation. For IL-6, significant differences were detected only after 24 h of stimulation. No differences in the responses of hPDLSCs and hGMSCs between “ultrapure” *P. gingivalis* LPS and “ultrapure” *E. coli LPS* were observed.

### 3.5. Effect of TLR-4 Inhibitor TAK-242 on the Response of Primary hPDLSCs and hGMSCs to Different LPS Preparations

**Figure 6:**
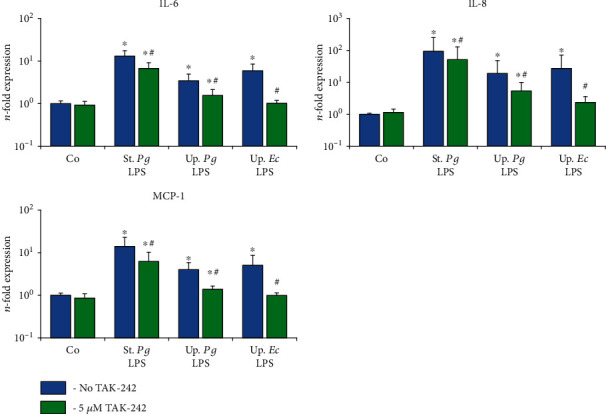
Effect of the TLR4 inhibitor TAK-242 on the gene expression levels of IL-6, IL-8, and MCP-1 in the hPDLSC response to different LPS preparations. Primary hPDLSCs were stimulated with commercially available “standard” *P. gingivalis* LPS (1 *μ*g/ml), “ultrapure” *P. gingivalis* LPS (1 *μ*g/ml), and “ultrapure” *E. coli* LPS (1 *μ*g/ml) in the presence (250 ng/ml) of sCD14. Additionally, in the appropriate groups, TLR4 inhibitor TAK-242 (5 *μ*M) was added for 24 h. The resulting gene expression levels of IL-6, IL-8, and MCP-1 were measured by qPCR. The *y*-axis represents the *n*-fold expression levels of target gene in relation to unstimulated cells (*n* = 1). Data are presented as mean ± s.e.m. of five different donors. ^∗^Significantly different *vs.* control; ^#^significantly different *vs.* stimulation in the absence of TAK-242.

**Figure 7:**
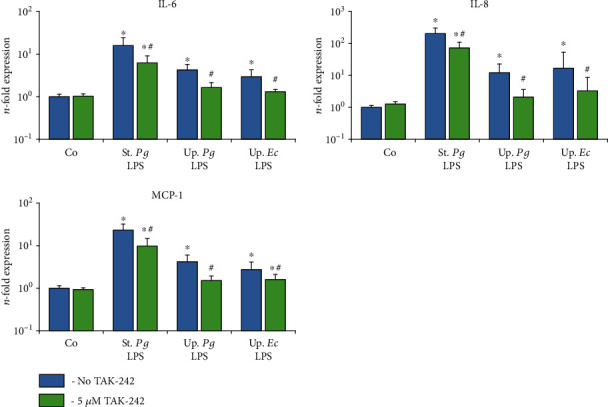
Effect of the TLR4 inhibitor TAK-242 on the gene expression levels of IL-6, IL-8, and MCP-1 in the hGMSC response to different LPS preparations. Primary hGMSCs were stimulated with commercially available “standard” *P. gingivalis* LPS (1 *μ*g/ml), “ultrapure” *P. gingivalis* LPS (1 *μ*g/ml), and “ultrapure” *E. coli* LPS (1 *μ*g/ml) in the presence (250 ng/ml) of sCD14. Additionally, in the appropriate groups, TLR4 inhibitor TAK-242 (5 *μ*M) was added for 24 h. The resulting gene expression levels of IL-6, IL-8, and MCP-1 were measured by qPCR. The *y*-axis represents the *n*-fold expression levels of target gene in relation to unstimulated cells (*n* = 1). Data are presented as mean ± s.e.m. of five different donors. ^∗^Significantly different *vs.* control; ^#^significantly different *vs.* stimulation in the absence of TAK-242.

**Figure 8:**
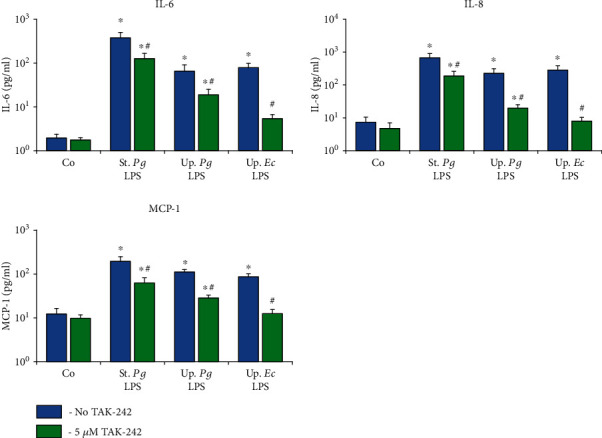
Effect of TLR4 inhibitor TAK-242 on concentrations of IL-6, IL-8, and MCP-1 in conditioned media of hPDLSCs upon stimulation with different LPS preparations. Primary hPDLSCs were stimulated with commercially available “standard” *P. gingivalis* LPS (1 *μ*g/ml), “ultrapure” *P. gingivalis* LPS (1 *μ*g/ml), and “ultrapure” *E. coli* LPS (1 *μ*g/ml) in the presence (250 ng/ml) of sCD14. In the appropriate groups, TLR4 inhibitor TAK-242 (5 *μ*M) was added for 24 h. The resulting protein concentrations of IL-6, IL-8, and MCP-1 were measured by ELISA in the conditioned media. Data are presented as mean ± s.e.m. of five different donors. ^∗^Significantly different *vs.* control; ^#^significantly different *vs.* stimulation in the absence of TAK-242.

**Figure 9:**
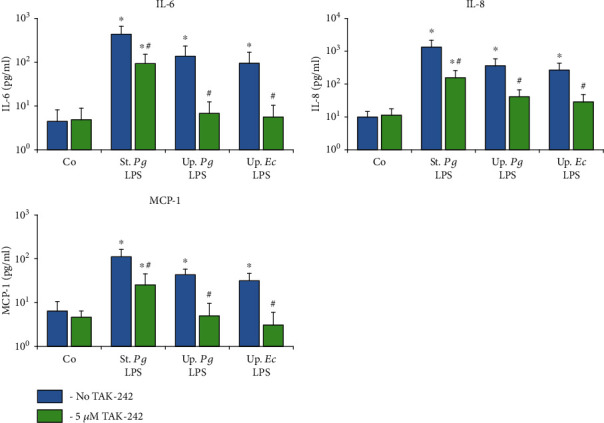
Effect of TLR4 inhibitor TAK-242 on concentrations of IL-6, IL-8, and MCP-1 in conditioned media of hGMSCs upon stimulation with different LPS preparations. Primary hGMSCs were stimulated with commercially available “standard” *P. gingivalis* LPS (1 *μ*g/ml), “ultrapure” *P. gingivalis* LPS (1 *μ*g/ml), and “ultrapure” *E. coli* LPS (1 *μ*g/ml) in the presence (250 ng/ml) of sCD14. In the appropriate groups, TLR4 inhibitor TAK-242 (5 *μ*M) was added for 24 h. The resulting protein concentrations of IL-6, IL-8, and MCP-1 were measured by ELISA in the conditioned media. Data are presented as mean ± s.e.m. of five different donors. ^∗^Significantly different *vs.* control; ^#^significantly different *vs.* stimulation in the absence of TAK-242.

The effect of the TLR-4 inhibitor TAK-242 on the gene expression levels of IL-6, IL-8, and MCP-1 in response to stimulation with the different LPS preparations was investigated for hPDLSCs (Figure 6) and hGMSCs (Figure 7) after 24 h of stimulation. The levels of corresponding proteins in conditioned media are presented in Figures 8 and 9, respectively. The response of hPDLSCs and hGMSCs to different bacterial LPS preparations was significantly inhibited by the TLR-4 inhibitor TAK-242. However, the degree of inhibition differed between “standard” and “ultrapure” LPS. As evaluated based on the protein production, TAK-242 inhibited the response of “standard” *P. gingivalis* by up to 73% in hPDLSCs and up to 83% in hGMSCs. In contrast, the response to “ultrapure” *P. gingivalis* LPS was inhibited by TAK-242 up to 93% in hPDLSCs and up to 100% in hGMSCs. The response to “ultrapure” *E. coli* LPS was inhibited by TAK-242 up to 98% in hPDLSCs and completely in hGMSCs.

### 3.6. Effect of Different LPS Preparations on TLR-2 and TLR-4 Expression in hPDLSCs and hGMSCs

**Figure 10:**
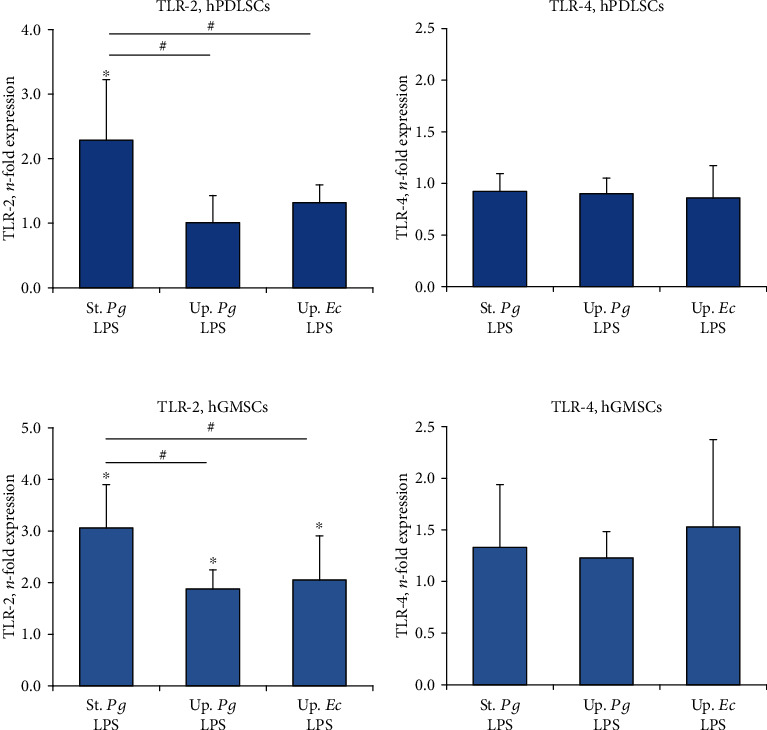
Effect of different LPS preparations on the expression of TLR-2 and TLR-4 in hPDLSCs and hGMSCs. Primary hPDLSCs and hGMSCs were stimulated with commercially available “standard” *P. gingivalis* LPS (1 *μ*g/ml), “ultrapure” *P. gingivalis* LPS (1 *μ*g/ml), and “ultrapure” *E. coli* LPS (1 *μ*g/ml) in the presence (250 ng/ml) of sCD14 for 24 h. Gene expression levels of TLR-2 and TLR-4 were measured by qPCR. The *y*-axis represents the *n*-fold expression levels of target gene in relation to unstimulated cells (*n* = 1). Data are presented as mean ± s.e.m. of five different donors. ^∗^Significantly different *vs.* control; ^#^significantly higher *vs.* “ultrapure” LPS preparations.

The effect of different LPS preparations on the gene expression of TLR-2 and TLR-4 in hPDLSCs and hGMSCs is shown in Figure 10. The expression of TLR-2 in hPDLSCs was significantly enhanced by “standard” *P. gingivalis* LPS but not affected by the “ultrapure” LPS preparations. In hGMSCs, the expression of TLR-2 was significantly increased by all investigated LPS preparations. In both cell types, the expression of TLR-2 in response to “standard” *P. gingivalis* LPS was significantly higher than that in response to “ultrapure” LPS preparations. The expression of TLR-4 was not affected by any LPS preparation in both cell types.

## 4. Discussion

In the present study, we investigated the effect of different *P. gingivalis* LPS preparations on the expression of IL-6, IL-8, and MCP-1 in hPDLSCs and hGMSCs. We found that “standard” *P. gingivalis* LPS induces a substantially stronger response in both cell types compared to “ultrapure” *P. gingivalis* LPS. These differences were especially pronounced after 24 h of stimulation, with “ultrapure” *P. gingivalis* LPS showing an about 10 times higher protein production compared to “standard” *P. gingivalis* LPS. At the same time, no significant differences between the response to “ultrapure” *P. gingivalis* LPS and *E. coli* LPS were observed in both cell types. In contrast to “standard” *P. gingivalis* LPS, in “ultrapure” *P. gingivalis* LPS, lipoproteins are enzymatically degraded and are not able to activate TLR2 and, therefore, we can conclude that these contaminations might substantially affect the cellular response to *P. gingivalis* LPS. Clear differences between “standard” and “ultrapure” LPS preparations were observed also in their ability to activate TLR-2 expression. In both cell types, TLR-2 expression was more strongly activated by “standard” *P. gingivalis* LPS than by both “ultrapure” LPS preparations. No differences were observed between the different “ultrapure” LPS originated from different bacterial species. Thus, our finding suggests that the response of hPDLSCs and hGMSCs to LPS depends on the purity of the LPS preparation rather than the LPS source.

According to the literature, “standard” *P. gingivalis* LPS is isolated by phenol-water extraction [37], containing about 2% of lipoprotein, about 1.5% of double-stranded DNA, and about 1.5% of RNA [45]. We have analyzed the composition of both commercially available *P. gingivalis* LPS preparations. A quantitative monosaccharide analysis revealed enrichment of LPS by approximately 40% in the “ultrapure” preparation compared to “standard” LPS. SDS gel electrophoresis analysis showed that the “standard” but not the “ultrapure” *P. gingivalis* preparation LPS contains silver-stained components in a molecular mass range between approximately 10 and 15 kDa (Supporting Figure 1). A comparable migration behavior of an LPS preparation was observed in a previous study, where on a silver-stained Tris/glycine gel, prominent *P. gingivalis* LPS bands were displayed in the range of 10-17 kDa, of which one band was confirmed by mass spectrometry to be *P. gingivalis* lipoprotein PG1828 [35]. Thus, based on these data and our direct comparison of the two LPS preparations of different purity grades on the gels within the frame of this study, it is likely that the visualized bands correspond to lipoproteins of *P. gingivalis*. If the blurred band below 10 kDa in the “ultrapure” LPS preparation corresponds to a low-abundance lipoprotein that is specifically retained in the “ultrapure” LPS preparation remains to be determined. Surprisingly, measurement of the protein concentration showed that the value for “ultrapure” *P. gingivalis* LPS is two times higher compared to that for “standard” *P. gingivalis* LPS, which approximates the enrichment factor of LPS in the “ultrapure” LPS preparation. It is conceivable to assume that the protein measured in the “ultrapure” sample originates from lipoproteins. These, however, have been proteolytically degraded during the purification procedure by the manufacturer and, hence, are not visible on the gel and no longer immunogenic. This is confirmed by the inability of “ultrapure” *P. gingivalis* LPS to activate TLR-2 reporter HEK-Blue hTLR2-hCD14 cells [39, 46].

Lipoproteins are potent TLR-2 agonists [47–50], leading to a half-maximal response *in vitro* already at concentrations of about 3 pM [51]. The lipoprotein concentration in commercially available “standard” LPS preparations is within this range [34]. Hence, it is conceivable that the lipoprotein-induced response triggered by TLR-2 potentiates the cellular response to “standard” LPS compared to “ultrapure” LPS. This is supported by data from Hashimoto et al. [35] showing that *P. gingivalis* lipoprotein PG1828 extracted from a commercially available “standard” *P. gingivalis* LPS preparation is a significantly stronger immunostimulator of human gingival fibroblasts than the lipid A moiety of *P. gingivalis* LPS. This is also in line with our previous studies showing that stimulating hPDLSCs with synthetic lipoprotein Pam3CSK4, a TLR-2 agonist, causes a significantly higher response compared to TLR-4 activation with *E. coli* LPS [18, 52, 53].

Interestingly, differences in the time dependency of the responses of both cell types to “standard” and “ultrapure” *P. gingivalis* LPS could be observed. In hPDLSCs, the gene expression levels of all investigated proinflammatory mediators were significantly higher after 24 h of stimulation with “standard” *P. gingivalis* LPS, compared to 4 h of stimulation. A similar trend was observed in hGMSCs, although the differences were not statistically significant. However, in hGMSCs, “standard” *P. gingivalis* LPS induced relatively high expression levels of all investigated proteins already after 4 h of stimulation. In contrast, no significant increase in the gene expression level after 24 h of stimulation compared to 4 h was observed for “ultrapure” LPS preparations in both hPDLSCs and hGMSCs. Moreover, the gene expression levels in response to “ultrapure” LPS after 24 h of stimulation were significantly lower than those after 4 h of stimulation. This finding suggests that in the investigated hPDLSCs and hGMSCs, contaminating lipoproteins do not only contribute to the intensity of the response but also induce prolongation of the response.

The TLR-4 inhibitor TAK-242 significantly reduced the amplitude of the response of hPDLSCs and hGMSCs to all LPS preparations. In both cell types, the response to *E. coli* LPS was significantly inhibited by TAK-242, similar to the unstimulated control. This finding is not surprising, since *E. coli* LPS is a well-known TLR-4 agonist [54, 55]. The response to “ultrapure” *P. gingivalis* LPS decreased in hGMSCs to a level comparable to that of unstimulated controls, which confirms the assumption that “ultrapure” *P. gingivalis* LPS activates a TLR-4 dependent response only. Surprisingly, in hPDLSCs, the response to “ultrapure” *P. gingivalis* LPS was not completely inhibited by TAK-242. Nevertheless, the response to “ultrapure” *P. gingivalis* LPS was inhibited more effectively than the response to “standard” *P. gingivalis* LPS. The residual response could be explained in two ways. First, TLR-4 might not have been completely inhibited by TAK-242. This assumption is confirmed by the fact that the response to “ultrapure” *E. coli* LPS in hPDLSCs was also slightly higher, although not statistically significant, compared to the control. Secondly, it is possible that the residual response to *P. gingivalis* LPS is accounted by TLR-2 activation. We could not prove this experimentally, because blocking of TLR-2 with specific anti-TLR-2 antibodies induced significant responses in hPDLSCs (unpublished observation). Previous studies by others showed that “ultrapure” *P. gingivalis* LPS triggers a response in both TLR-2- and TLR-4-expressing cells [31, 56]. While we have not obtained experimental evidence of the presence of typical *P. gingivalis* lipoproteins in the “ultrapure” LPS preparation, it cannot be ruled out that low-abundance, biologically active lipoproteins are still contained in the preparation which might have escaped from detection. This still leaves the option of any TLR-2 activation of *P. gingivalis* LPS being due to lipoprotein contamination. However, even if some differences in the hPDLSC response to LPS of different bacterial species exist, they are markedly lower than those caused by *P. gingivalis* LPS of different purity.

In contrast to “ultrapure” LPS preparations, the response to “standard” *P. gingivalis* LPS was not that effectively inhibited by TLR-4 inhibitor TAK-242. This observation is not surprising, given that contaminating lipoproteins can trigger a TLR-2 dependent response [34, 35, 39, 47–50]. An interesting observation of our study is that in absolute values, the response to “standard” *P. gingivalis* LPS upon TAK-242 inhibition was markedly higher than that to “ultrapure” *P. gingivalis* LPS. This might suggest synergistic effects by simultaneous activation of TLR-2 and TLR-4 receptors. Several studies already demonstrated a synergy in the production of cytokines by conventional dendritic cells [57] or a synergistic upregulation of a scavenger receptor in macrophages [58] via simultaneous TLR-2 and TLR-4 signaling. However, the relevance of these mechanisms in hPDLSCs and hGMSCs needs to be further investigated. Nevertheless, independently on the underlying mechanisms, we can conclude that the response of MSC-like cells derived from periodontal tissue is affected drastically by the presence of minor contaminations. This might be an explanation of the contradictory results found in different experimental studies for the immunogenicity of *P. gingivalis* LPS in the literature [26, 31, 36, 39, 59–61]. Attributing the effect of LPS to contaminating lipoproteins can lead to misinterpretations of the role of *P. gingivalis* LPS in the inflammatory response. An important limitation of this study is that we used LPS from only one supplier. The procedure of LPS isolation and purification might differ between different LPS suppliers, which can affect the cellular response to LPS qualitatively and quantitatively. However, the purity of LPS preparations is an important aspect to consider, when studying cell responses *in vitro.*

Our previous studies on hPDLSCs [18, 52, 53] and a study of other groups on gingival cells [35] showed that TLR-2 agonists induce a much stronger proinflammatory response than TLR-4 agonists, which suggests that TLR-2 might play an important role in periodontal tissue destruction. It is known that TLR-4 and TLR-2 expression levels are highly increased in periodontitis lesions, compared to healthy periodontal ligament [62]. Several studies showed that TLR-2 expression levels are associated with the inflammation severity of the periodontium [63–68]. Furthermore, the essential role of TLR-2 in periodontitis-associated tissue destruction was demonstrated in multiple *in vivo* studies. Several animal studies with different periodontitis models showed that TLR-2-deficient mice exhibit lower levels of alveolar bone loss and proinflammatory cytokine expression compared to wild-type and TLR-4-deficient mice [69–72]. An excessive response of resident tissue cells to a TLR-2 agonist compared to a TLR-4 agonist might also partially contribute to the differences observed between TLR-2- and TLR-4-deficient mice. It should be mentioned that other studies reported a significantly higher bone loss and a more severe degree of periodontitis in TLR-2-deficient mice than in wild-type mice [73, 74]. This can be explained by using specific mouse strains and their variable genetic constitutions [75]. Nevertheless, the exact role of TLR-2 in the pathogen elimination on the one hand and in the excessive inflammatory response on the other hand needs further investigation.

The dependency of the response on LPS source and purity was largely similar in both hPDLSCs and hGMSCs; however, minor differences between these two cell types could be observed. Firstly, the differences in the response between “standard” and “ultrapure” *P. gingivalis* LPS were observed in hPDLSCs only after 24 h stimulation, whereas in hGMSCs, already after 4 h. Secondly, in hPDLSCs, only “standard” *P. gingivalis* LPS induced TLR-2 expression, whereas in hGMSCs, it was induced also by “ultrapure” LPS. Thirdly, as mentioned above, in hPDLSCs but not in hGMSCs, some resting significant response to “ultrapure” LPS was observed even in the presence of TAK-242. One reason for these differences could be various anatomical positions of gingival and periodontal ligament tissues. The periodontal ligament is less likely to be exposed to bacterial stimuli than gingival tissue, which might result in a different susceptibility of hPDLSCs and hGMSCs to bacterial components. Furthermore, hPDLSCs and hGMSCs might have slightly different expression levels of various proteins, involved in TLR-mediated response. However, the exact differences between these two cell types need to be investigated in especially designed studies.

Although we did not observe substantial differences in the responses between the “ultrapure” LPS preparations from *P. gingivalis* and *E. coli* within the frame of this study, it needs to be mentioned that the difference in their lipid A composition is known to have biological implications. Lipid A of *P. gingivalis* is either tetra- or pentaacetylated, which is in contrast to hexaacetylated *E. coli* LPS [33]. This structural difference is associated with different inflammatory responses. Moreover, the structure of *P. gingivalis* LPS can be influenced by external factors, like nutrients availability and temperature [76, 77], which might partially account for the ability of this keystone pathogen to subvert the host immune system [33]. Previous studies, using highly purified *P. gingivalis* preparations (less than 0.1% of contaminating proteins), support the critical role of lipid A composition in the response of human gingival fibroblasts. Notably, *P. gingivalis* LPS with pentaacetylated lipid A induced markedly higher activation of NF-*κ*B and production of IL-6 and IL-8 compared to *P. gingivalis* LPS with tetraacetylated lipid A [26, 78]. With regard to the present study, a different lipid A composition of the two LPS preparations from *P. gingivalis* can be excluded, since they originate from the same source. It will be an important aspect of future studies to evaluate the role of *P. gingivalis* LPS purity in combination with LPS composition.

The clinical relevance of our study is limited due to its *in vitro* design. The *in vivo* situation is very complex and involves the interaction of several cell types with numerous bacterial virulence factors. Therefore, we assume that the “standard” LPS preparation reflects the *in vivo* situation more adequately compared to the “ultrapure” preparation. Our results show a significantly higher immunogenicity of “standard” *P. gingivalis* LPS, which is contaminated with other bacterial components, whereas the immunogenicity of “ultrapure” LPS preparation was relatively low. Therefore, our data suggest that rather the simultaneous exposure of local cells of the periodontal tissue to several virulence factors and their response to these factors might play a more essential role in the progression of periodontal disease than the response to LPS itself. This might be an important perspective for further understanding the mechanisms of the inflammatory response in periodontitis and the development of future treatment modalities.

## 5. Conclusion

In conclusion, our study shows that the purity of an LPS preparation plays a more important role in the response of MSCs from periodontal tissue than the LPS source. Even rather small contaminations (less than 2%) with lipoproteins, dsDNA, or RNA can substantially enhance the response of both investigated cell types to *P. gingivalis* LPS. The procedure of LPS preparation is undoubtedly a critical factor, which must be considered when interpreting results and comparing different studies. Furthermore, the contribution of *P. gingivalis* LPS to the progression of periodontitis should be only considered in connection with other bacterial components, which might drastically change the cellular host response to this virulence factor.

## Data Availability

The data used to support the findings of this study are available from the corresponding author upon request.

## Supplementary Materials

Supplementary Materials Supplementary Figure 1: SDS gel electrophoresis of “standard” and “ultrapure” P. gingivalis LPS preparations, followed by silver staining. Supplementary Table 1: hPDLSC and hGMSC mesenchymal and hematopoietic surface marker expression analysis.
