# Supplementary material for: Response of Human Mesenchymal Stromal Cells from Periodontal Tissue to LPS Depends on the Purity but Not on the LPS Source
Source: Mediators Inflamm. 2020 Jul 2;2020:8704896. doi: 10.1155/2020/8704896 (PMC7352132; doi:10.1155/2020/8704896)
Supplement: Supplementary Materials — Supplementary Figure 1: SDS gel electrophoresis of “standard” and “ultrapure” P. gingivalis LPS preparations, followed by silver staining. Supplementary Table 1: hPDLSC and hGMSC mesenchymal and hematopoietic surface marker expression analysis. [file 8704896.f1.docx]

**Supplementary Material**

***Response of human mesenchymal stromal cells from periodontal tissue to LPS depends on the purity but not on the LPS source***

Christian Behm^1^, Alice Blufstein^1^, Setareh Younes Abhari^1^, Christoph Koch^1^, Johannes Gahn^1^, Christina Schäffer^2^, Xiaohui Rausch-Fan^1^, Oleh Andrukhov (OA)^1^

^1^Division of Conservative Dentistry and Periodontology, University Clinic of Dentistry, Medical University of Vienna, Vienna, Austria

^2^Department of NanoBiotechnology / *NanoGlycobiology* Unit, University of Natural Resources and Life Sciences, Vienna, Austria

Correspondence should be addressed to Oleh Andrukhov; oleh.andrukhov@meduniwien.ac.at


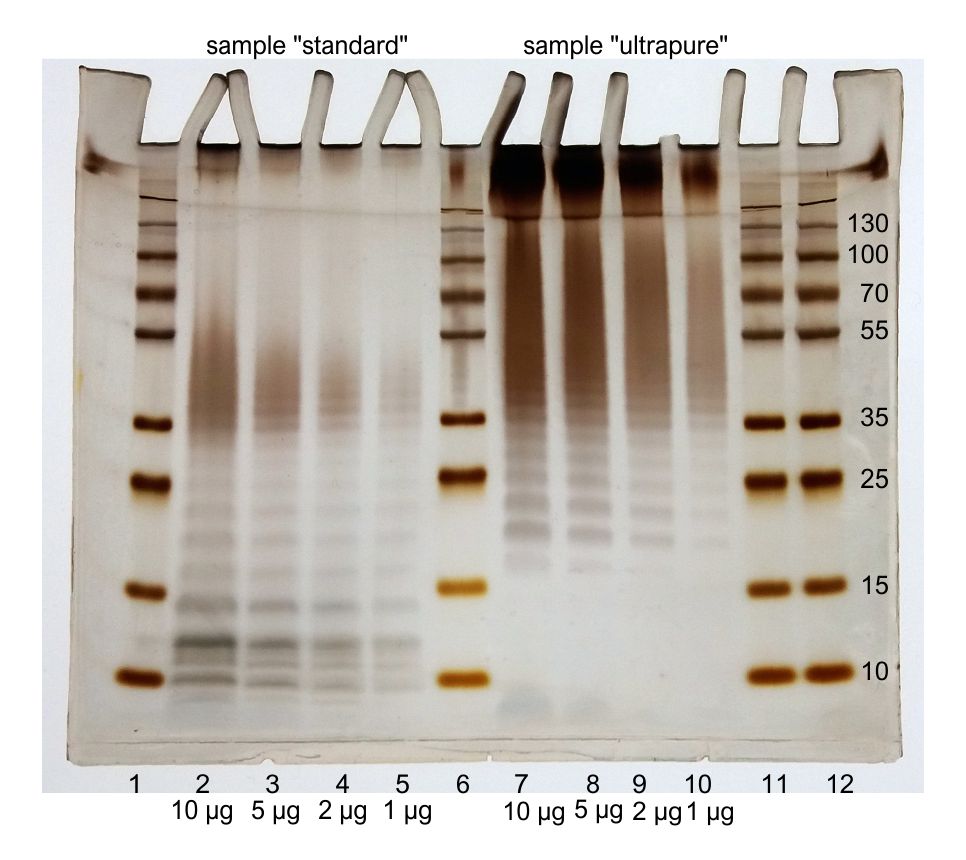
**Supplementary Figure 1**

**Supplementary Figure 1.** SDS gel electrophoresis of “standard” and “ultrapure” *P. gingivalis* LPS preparations, followed by silver staining. 1 (lanes 5, 10), 2 (lanes 4, 9), 5 (lanes 3, 8) and 10 µg (lanes 2, 10) of “standard” and “ultrapure” *P. gingivalis* LPS, respectively, were loaded on a 8-16% Tris/Tricine TGS precast gel followed by silver staining for visualization of LPS and protein content. Prestained protein-marker V, peqGOLD (VWR, Vienna, Austria) was used as standard (molecular masses given in kDa).

**Supplementary Table 1**

|  |  | hPDLSCs | | | hGMSCs | | | |  | |
| --- | --- | --- | --- | --- | --- | --- | --- | --- | --- | --- |
|  | | | MSC Marker | Hematopoietic Marker | MSC Marker | Hematopoietic Marker | | | |  |
| CD29 | | | 97.7 ± 0.2 |  | 98.7 ± 0.4 | |  | | |  |
| CD73 | | | 96.1 ± 0.2 |  | 98.7 ± 0.4 | |  | | |  |
| CD90 | | | 97.9 ± 0.2 |  | 98.4 ± 0.8 | |  | | |  |
| CD105 | | | 97.1 ± 0.6 |  | 97.9 ± 1.3 | |  | | |  |
| CD146 | | | 61.3 ± 5.7 |  | 40.2 ± 12.7 | |  | | |  |
|  | | |  |  |  | | |  | |  |
| CD31 | | |  | 0.5 ± 0.1 |  | | | 0.5 ± 0.0 | |  |
| CD34 | | |  | 0.6 ± 0.2 |  | | | 2.0 ± 0.2 | |  |
| CD45 | | |  | 2.7 ± 0.2 |  | | | 2.5 ± 0.3 | |  |

**Supplementary Table 1.** hPDLSCs and hGMSCs mesenchymal and hematopoietic surface marker expression analysis. Data are presented as mean values ± s.e.m. from five experiments using cells from five different donors.
